# Influence of Glycerol on the Surface Morphology and Crystallinity of Polyvinyl Alcohol Films

**DOI:** 10.3390/polym16172421

**Published:** 2024-08-27

**Authors:** Ganna Kovtun, David Casas, Teresa Cuberes

**Affiliations:** 1 Institute of Magnetism NAS of Ukraine and MES of Ukraine, 03142 Kyiv, Ukraine; 2 Group of Nanotechnology and Materials, Mining and Industrial Engineering School of Almaden, University of Castilla-La Mancha, 13400 Almaden, Spain; david.casas@uclm.es (D.C.); teresa.cuberes@uclm.es (T.C.)

**Keywords:** polyvinyl alcohol films, PVA/glycerol blends, crystallinity, infrared spectroscopy, X-ray diffraction, thermogravimetry, differential thermal analysis, atomic force microscopy, lateral force microscopy, ultrasonic force microscopy

## Abstract

The structure and physicochemical properties of polyvinyl alcohol (PVA) and PVA/glycerol films have been investigated by Fourier transform infrared spectroscopy (FT-IR), X-ray diffraction (XRD), thermogravimetry/differential thermal analysis (TG/DTA), and advanced scanning probe microscopy (SPM). In the pure PVA films, SPM allowed us to observe ribbon-shaped domains with a different frictional and elastic contrast, which apparently originated from a correlated growth or assembly of PVA crystalline nuclei located within individual PVA clusters. The incorporation of 22% *w*/*w* glycerol led to modification in shape of those domains from ribbon-like in pure PVA to rounded in PVA/glycerol 22% *w*/*w* films; changes in the relative intensities of the XRD peaks and a decrease in the amorphous halo in the XRD pattern were also detected, while the DTA peak corresponding to the melting point remained at almost the same temperature. For higher glycerol content, FT-IR revealed additional glycerol-characteristic peaks presumably related to the formation of glycerol aggregates, and XRD, FT-IR, and DTA all indicated a reduction in crystallinity. For more than 36% *w*/*w* glycerol, the plasticization of the films complicated the acquisition of SPM images without tip-induced surface modification. Our study contributes to the understanding of crystallinity in PVA and how it is altered by a plasticizer such as glycerol.

## 1. Introduction

Nowadays, the development of materials with film-forming capacity is of growing interest. Polyvinyl alcohol (PVA) is one of the most popular synthetic polymers due to its biodegradability, biocompatibility, high elasticity, tribological response, hydrophilicity, and acceptable thermal properties. PVA has excellent film-forming properties and is widely used in the development of sensor materials, drug delivery systems, wound dressings, artificial cartilages, implants, for packaging applications, recovery of organic and inorganic pollutants, etc. [1,2,3,4,5,6].

However, due to its poor ductility, in practical applications PVA is often used with the addition of plasticizers, which influence its mechanical and thermodynamic properties [7,8,9]. Plasticizers such as glycerol, sorbitol, sucrose, and some organic acids are well-known as low-molecular-weight compounds that reduce polymer–polymer chain bonding [10]. The breaking of hydrogen bonds increases the molecular mobility of the polymer, which results in increased flexibility of the polymer films. Being nontoxic to humans at low concentrations, glycerol is a widespread plasticizer incorporated in PVA films [11,12,13,14,15,16,17,18,19], as well as in its nanocomposites and blends.

PVA is a semicrystalline polymer composed of both amorphous and crystalline phases, with the percentage of crystallinity playing a crucial role in determining its performance. Understanding and controlling the crystallinity of PVA is essential for applications such as drug delivery and wound dressings, as the amount of the crystalline phase will affect the release of active compounds and the material’s mechanical properties. Higher crystallinity may enhance the durability and structural integrity of films for biomedical applications. Additionally, in films used as membranes for water treatment and the recovery of organic and inorganic pollutants, crystalline regions can improve mechanical strength and chemical resistance, leading to better performance and longer service life. For adhesives and coatings, the crystallinity of PVA affects adhesion properties, flexibility, and resistance to solvents and environmental factors. In packaging, it influences the film’s strength, barrier properties, and stability. Controlling crystallinity can help optimize these films for specific industrial purposes.

In this work, we have conducted a comprehensive analysis of the crystallinity in pure PVA and PVA/glycerol films through a correlative study of data from fast Fourier transform infrared spectroscopy (FT-IR), X-ray diffraction (XRD), thermogravimetry (TG)/differential thermal analysis (DTA), and advanced scanning probe microscopy (SPM).

The use of atomic force microscopy (AFM), friction force microscopy (FFM), and ultrasonic force microscopy (UFM) enables us to map the topographical features of these films, as well as their frictional and mechanical responses, at the nanoscale. UFM is a relatively new AFM-based technique that provides contrast based on nanoscale differences in surface or near-subsurface sample stiffness and adhesive properties [20]. To the best of our knowledge, this is the first time an extensive scanning probe microscopy (SPM) study of this nature has been conducted on these films. SPM offers a unique platform for analyzing the surface properties of polymer films at the nanoscale. It not only allows for detailed examination of surface morphology but also facilitates the investigation of frictional and mechanical characteristics through direct interaction with surface atoms. Unlike scanning electron microscopy, which typically requires ultra-high vacuum conditions and involves the interaction of an electron beam with the polymer surface, SPM studies can be conducted in ambient conditions. This makes SPM a versatile and practical tool for these investigations. By additionally integrating data for structural, mechanical, physicochemical, and thermal characterization from the aforementioned experimental techniques, we seek to achieve a detailed understanding of how glycerol influences the crystallinity and overall behavior of PVA film.

Our study discloses valuable insights into how plasticizers, such as glycerol, impact the PVA nanostructure, thereby offering avenues for tailoring PVA properties to achieve optimal performance for specific applications.

## 2. Materials and Methods

### 2.1. Materials and Sample Preparation

**Figure 1:**
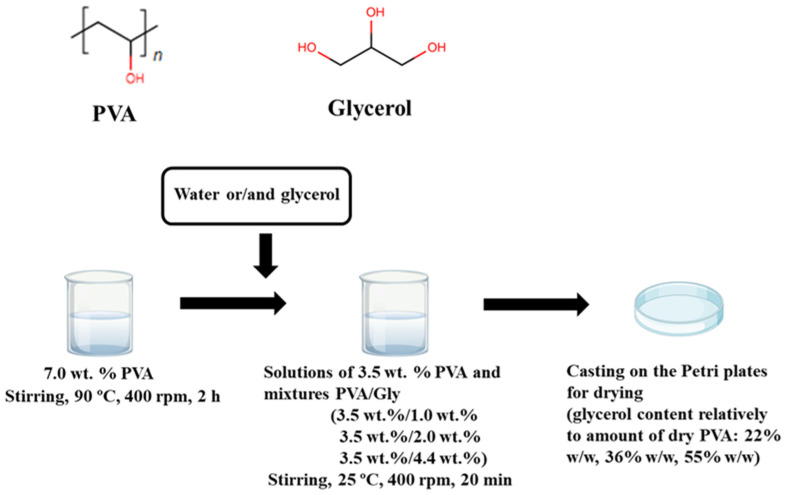
Structural formulas of PVA and glycerol; film preparation scheme.

PVA (Mw 31,000–50,000 g/mol, 98–99% hydrolyzed) and glycerol (≥99.0%) were supplied by Merck, Darmstadt, Germany. The PVA was dissolved in distilled water with stirring at 90 °C, 400 rpm, when heated in a water bath for 2 h, to prepare a 7.0 wt.% stock solution. Solutions of PVA with 3.5 wt.% concentration and mixed PVA/glycerol solutions containing 3.5 wt.% PVA and 1.0 wt.%, 2.0 wt.%, and 4.4 wt.% glycerol were prepared from the 7.0 wt.% PVA stock solution, glycerol, and distilled water, stirred at 25 °C, 400 rpm, 20 min, poured on polystyrene Petri dishes, and evaporated at room temperature (20–25 °C) and ambient humidity ~50% R.H. The glycerol content in the prepared films in relation to the amount of dry PVA was 22% *w*/*w*, 36% *w*/*w*, and 55% *w*/*w*. Films ~200 µm thick were obtained by this method (solution casting). The film preparation procedure is illustrated in Figure 1.

### 2.2. Fourier-Transformed Infrared Spectroscopy (FT-IR)

FTIR spectra (4 cm^−1^ resolution, wavenumber range 500–4000 cm^−1^) were recorded with a Shimadzu IRPrestige-21 spectrometer (Tokio, Japan), using the ATR method. Small pieces of the PVA and PVA/glycerol films were cut and placed in the instrument sample holder. The data were acquired using the software Shimadzu IR Solution 1.21 (Tokio, Japan) and analyzed using Originpro 2024 (Northampton, MA, USA) software.

### 2.3. X-ray Diffraction (XRD)

XRD measurements were performed in an equipment Philips X’Pert MPD (Eindhoven, Holland) using CuK α radiation (1.54056 Å) with 40 KV and 40 mA. It incorporates 0.04 rad soller slits for both incident and diffracted beams, an automatic 12.5 mm programmable divergence slit, and a Xe gas-sealed proportional detector. Data were collected in an angular range between 1° and 50° (2θ) with a step size of 0.02° and a counting time of 0.70 s per step, and analyzed using Originpro 2024 (Northampton, MA, USA) software.

### 2.4. Thermogravimetric Analysis (TGA) and Differential Thermal Analysis (DTA)

The thermal behavior of the samples was examined with a Setaram model TG/DTA92 equipment (Caluire-et-Cuire, France) using a Pt crucible. Typically, thermograms were recorded in air atmosphere, within a temperature range from 20 °C to 500 °C, with a heating rate of 25 °C/min. The data were analyzed using Originpro 2024 (Northampton, MA, USA) software.

### 2.5. Scanning Probe Microscopy

Contact-mode atomic force microscopy (AFM), lateral force microscopy (LFM), and ultrasonic force microscopy (UFM) were performed using a NANOTEC (Madrid, Spain) instrument. The modification of the AFM equipment for the incorporation of UFM facilities is described in [20]. For UFM, ultrasonic frequencies of ~3.8 MHz and modulation frequencies of 2.4 KHz were applied from a piezoelectric element placed under the sample. Typically, Olympus silicon nitride cantilevers with a nominal spring constant of 0.06 N/m and a nominal tip radius of 20 nm were used. The measurements were performed in air at ambient conditions (20–25 °C, ~50% R.H.). Data were analyzed using the WSxM 5.0 software (Madrid, Spain) [21].

## 3. Results and Discussion

**Figure 2:**
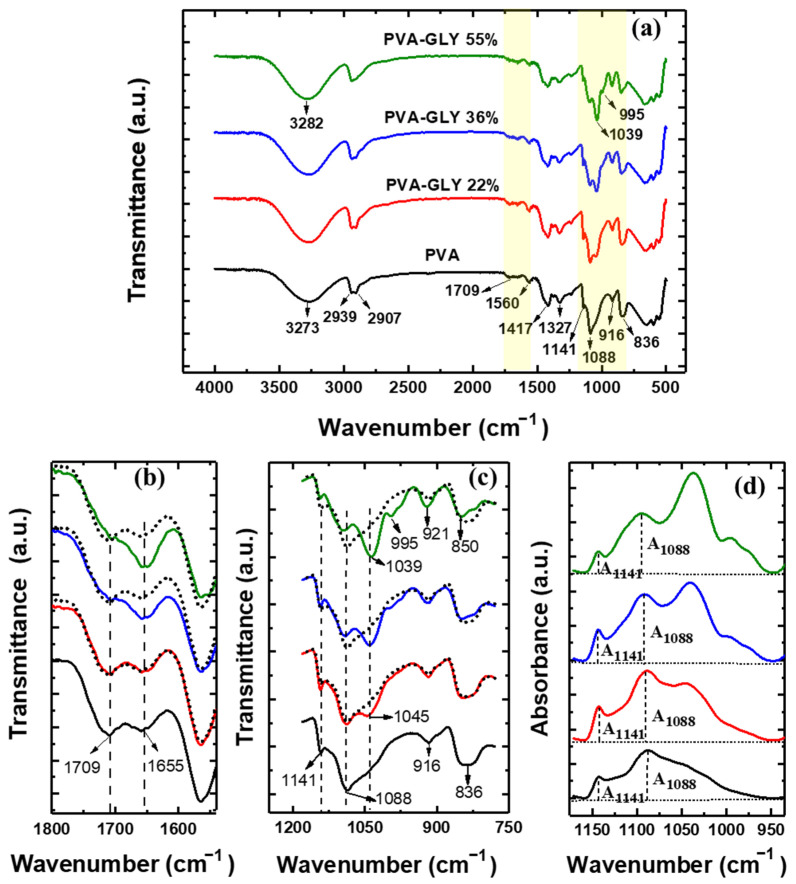
(**a**) Normalized FT-IR spectra of the pure PVA (black curve) and PVA/glycerol films with different concentrations of glycerol: 22 (red curve), 36 (blue curve), 55 (green curve) % *w*/*w*. (**b**,**c**) Zoom of regions highlighted in yellow in (**a**). In (**b**,**c**), the dotted black curves correspond to the pure PVA film, which has been shifted and superimposed on each of the other curves to facilitate comparison. (**d**) Absorbance spectra (inverse from the transmittance) in the selected region to illustrate the procedure for constructing the baseline to determine *A*_1141_ and *A*_1088_ in Equation (1) (see text).

FTIR spectra for the pure PVA and PVA/glycerol blended films with different glycerol contents (22, 36, and 55% *w*/*w*) are displayed in Figure 2a.

For the pure PVA film, the band at 3273 cm^−1^ corresponds to (O-H) stretching vibration from the intermolecular and intramolecular hydrogen bonds [18]. The two bands at 2939 and 2907 cm^−1^ correspond to the asymmetric and symmetric stretching vibrations of methylene (–CH_2_–), respectively [22]. The peaks at 1709 cm^−1^, 1655 cm^−1^, and 1560 cm^−1^ have been related to the stretching vibrations of the (C=O) and (C–O) bonds present in the remaining acetate units [23], which in our case must be very few, as we are using highly hydrolyzed PVA. The 1655 cm^−1^ band has also been assigned to absorbed water [24,25]. The peaks at 1417 cm^−1^ and 1327 cm^−1^ have been attributed to bending vibrations of hydroxyl (–OH) and wagging of (C–H), respectively [26]. The peak at 1141 cm^−1^ has been assigned to (C–O) stretching vibrations in C–OH groups of the crystalline polymer phase [27] and also to (C–C) stretching vibrations of the carbon framework of the polymer chain in the crystalline phase [28]. The peak at 1088 cm^−1^ corresponds to the (C–O) stretching vibrations [25], the band at 916 cm^−1^ to CH_2_ rocking vibration, and this at 836 cm^−1^ to (C–C) stretching and (C–H) out-of-plane vibrations.

For the PVA-glycerol films, (Figure 2a) a shift of the (O–H) stretching vibration band from 3273 cm^−1^ to higher wavenumbers is observed when increasing the glycerol content (up to 3283 cm^−1^ for 55% *w*/*w* of glycerol). A possible reason for this effect is the dissociation of hydrogen bonds between the PVA chains and the formation of new hydrogen bonds between the PVA and glycerol molecules [18,29]. Similar results were obtained for films of PVA/chitosan with the addition of glycerol [29] and films of wheat starch with glycerol [30].

For the PVA/glycerol 55% *w*/*w* film, the band at 2939 cm^−1^ experiences a slight increase compared to the 2907 cm^−1^ band. For pure glycerol, the aliphatic (C–H) group bands appear at 2931 cm^−1^ and 2879 cm^−1^. Hence, the aggregation of glycerol molecules within the PVA matrix might possibly account for this effect [31,32].

The peak at 1655 cm^−1^ increases with increasing glycerol concentration (see Figure 2b). A similar effect is noticeable from the FT-IR spectra for PVA/glycerol films in [13], although the origin of this effect was not discussed in this reference. According to [31] an FT-IR band at ~1653 cm^−1^ appears for commercial glycerol. This band may be assigned to (H–O–H) bending vibrations of water. Hence, the observed increase in the peak at ~1653 cm^−1^ with increasing glycerol content (see Figure 2b) may be indicative of an increase in the amount of water in the film.

The most noticeable change in the glycerol-modified PVA films is the appearance of a new peak at 1039 cm^−1^ (see Figure 2a,c).

In the FT-IR spectrum of pure glycerol, five characteristic bands have been observed, located at 800 up to 1150 cm^−1^ corresponding to vibrations of (C–C) and (C–O) linkages: three broad bands at 850, 925, and 995 cm^−1^ that have been attributed to the vibration of the skeleton (C–C); a peak at 1045 cm^−1^ associated to the stretching of the (C–O) linkage in the primary alcohol groups and a band at 1117 cm^−1^ that corresponds to the stretching of (C–O) in the secondary alcohol group [32,33,34].

In Figure 2c, a peak at 1045 cm^−1^ appears for the film with 22% *w*/*w* glycerol content (red curve) and increases in intensity while slightly displacing to lower wavelengths, up to 1039 cm^−1^, as the amount of glycerol incorporated into PVA is increased (blue and green curves). Such peak corresponds to (C–O) stretching of the primary alcohol groups of glycerol, and its shifting to lower wavelengths is indicative of increased glycerol/PVA interactions—presumably of the hydrogen bonding type—for higher glycerol contents. For PVA films with 36 and 55% *w*/*w* glycerol (blue and green curves in Figure 2c,d), the PVA band at 1088 cm^−1^ shows a clear modification of its shape due to an increase in intensity at ~1117 cm^−1^ (corresponding to stretching of (C–O) in the secondary alcohol group of glycerol), and slightly displaces to higher wavelengths (1091 and 1093 cm^−1^ for 36 and 55% *w*/*w* glycerol, respectively).

The fact that for 22% *w*/*w* glycerol the characteristic peak of glycerol for the primary alcohol group appears at 1045 cm^−1^, as in pure glycerol, suggests that for that film (22% *w*/*w* glycerol), it is the secondary alcohol group that preferentially forms hydrogen bonds with the PVA molecules. As the amount of glycerol is increased, additional types of glycerol interactions within the PVA matrix may take place, and the peak corresponding to the primary alcohol group displaces to lower wavelengths, up to 1039 cm^−1^.

For the 36% *w*/*w* glycerol films, the emergence of a band at ~995 cm^−1^ is also apparent, indicative of the excitation of vibrations of the glycerol (C–C) skeleton. For the film with 55% *w*/*w* glycerol, four glycerol bands are apparent in the covered wavelength range in Figure 2c. In addition to bands at 1039 and 995 cm^−1^, the intensity of the band at 921 cm^−1^ increases, and a clear peak at 850 cm^−1^ appears, while for pure PVA a relatively broad band at 836 cm^−1^ is observed (Figure 2c).

According to the literature [25,35], the crystallinity of various PVA films can be calculated from the FT-IR data by quantitative correlation between the intensities of the bands corresponding to the crystalline and amorphous phases. The positions of the PVA crystallinity absorption maximum given in the literature vary in the range 1141–1145 cm^−1^. We have used the value 1141 cm^−1^ that corresponds to the absorption maximum obtained in our experiments to calculate the crystallinity α of our PVA and PVA/glycerol films and considered the peak at 1088 cm^−1^ attributed to (C–O) stretching vibration as representative of the amorphous phase, according to the equation [25]:

(1) $$\propto \;=-13.1+89.5\times \left(\frac{A_{1141}}{A_{1088}}\right)$$

where *A*_1141_ and *A*_1088_ are the intensities of the absorption peaks in the crystalline and amorphous phases, respectively. According to [25], this equation is applicable for the calculation of α values in the range 18–60% with a correlation coefficient of 0.999 and an absolute error of determining α ± 0.3%.

The detailed procedure for constructing the baseline relative to which the intensities of the peaks *A*_1141_ and *A*_1088_ are to be measured is described in [25], and it is illustrated in Figure 2d. The baseline must pass through the minima in the absorption spectrum located at the edges of the examined spectral range, as depicted in Figure 2d. The position of the (C–O) band maximum at 1088 cm^−1^ varied slightly with increasing glycerol concentration, 1091 and 1093 cm^−1^ for PVA/glycerol 36 and 55% *w*/*w*, respectively (for sake of clarity, we refer to it as the band at 1088 cm^−1^ in all cases). The crystallinity band maximum is almost at the same position for the different films: 1141 cm^−1^ for the pure PVA film and 1142 cm^−1^ for PVA/glycerol 55% *w*/*w*.

The crystallinity of the PVA films with different glycerol content calculated by FT-IR is shown in Table 1. It is apparent that the crystallinity first increases with the addition of glycerol and then decreases to a level below that of the pure PVA film. Possibly, a small amount of glycerol, with their secondary alcohol groups linked by hydrogen bonding to the PVA chains, may even locally enhance their regular arrangements, resulting in an increase in PVA crystallinity. As the glycerol content increases, more glycerol molecules are available to interact with the PVA or with each other, destabilizing the interactions between the PVA chains and thus reducing the crystallinity.

The influence of glycerol on the crystalline structure of PVA films prepared via solution casting was also investigated by means of XRD. According to [36,37], the XRD diffractogram of pure PVA presents characteristic peaks at 2θ ~11.2° (100), 16.1° (001), 19.4° and 20.1° $\left(10\bar{1}\right)$/(101), and 22.5° (200). A crystalline peak at ~40–42° related to diffraction from $\left(1\bar{1}1\right)$/(210) planes has also been reported [37,38,39].

**Figure 3:**
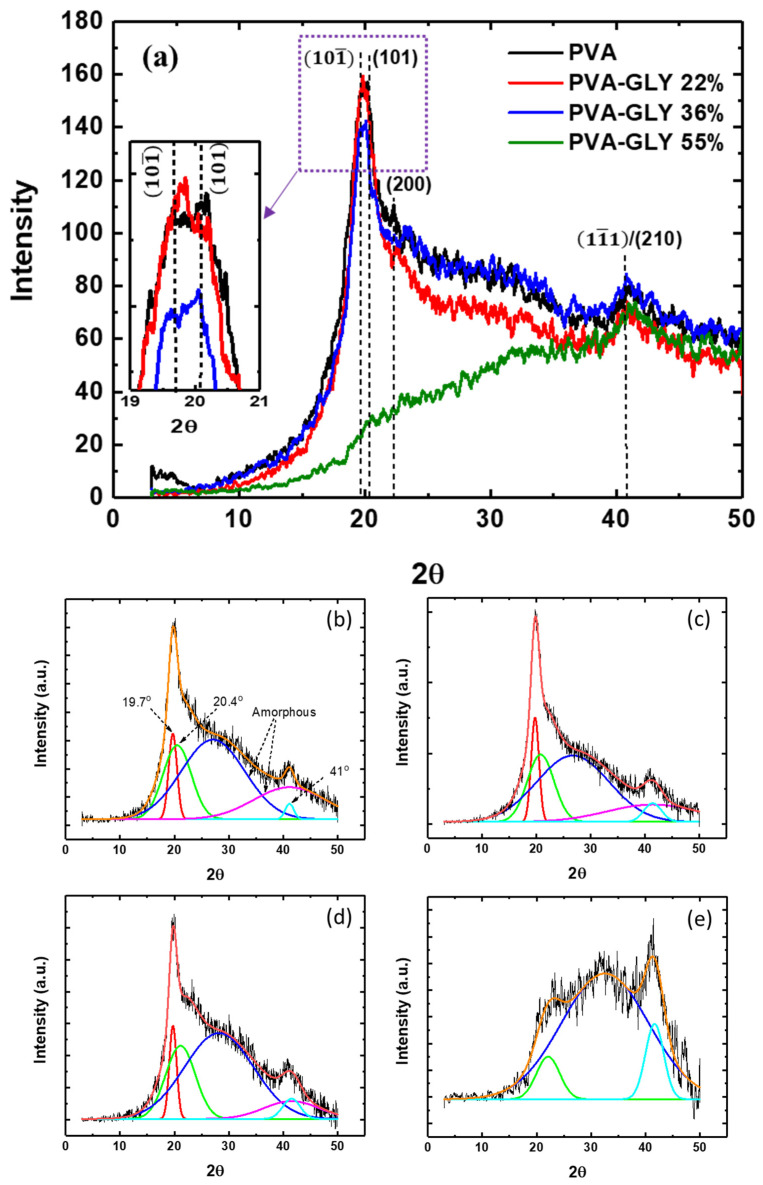
(**a**) XRD patterns of pure PVA (black curve) and PVA/glycerol films with different glycerol content: 22 (red curve), 36 (blue curve), 55 (green curve) % *w*/*w*. The inset displays an enlarged region of the peak at approximately 20° for pure PVA (black curve) and PVA/glycerol films with different glycerol content: 22 (red curve), 36 (blue curve) % *w*/*w*. (**b**–**e**) Fitting of XRD curve for PVA (**b**), PVA/glycerol 22% *w*/*w* (**c**), PVA/glycerol 36% *w*/*w* (**d**), PVA/glycerol 55% *w*/*w* (**e**).

Figure 3 shows the XRD diffractograms of the pure PVA and PVA/glycerol films with different glycerol content. The expected positions for the (100), (001), $\left(10\bar{1}\right)$/(101), and (200) diffraction peaks are indicated by dashed lines. For the films of pure PVA, and those with 22 and 36% *w*/*w* glycerol content, a diffraction peak about 2θ = 19.7° is clearly distinguished, as well as a small hump at ~40.7°. The obtained XRD patterns are typical for a semicrystalline polymer, with a broad amorphous halo originating from disordered (noncrystalline) regions. We understand that broad amorphous peaks (halos) in the XRD diffractogram reflect preferential interatomic distances, corresponding to the maximum of the amorphous halo, within the disordered regions of the polymer. From Figure 3, it can be seen that, as a result of the addition of 22% *w*/*w* glycerol (red curve in the figure), the diffuse scattering associated with the amorphous phase is reduced, which may be due to a structural rearrangement of the polymer chains. In [17], it was suggested that the introduction of glycerol in PVA films could significantly influence the structure of the amorphous domains. According to the FT-IR data, the crystallinity of this film experiences a slight increase (see Table 1). For a glycerol concentration of 36% *w*/*w* (blue curve), the XRD diffractogram shows now a larger contribution of the amorphous phase, similar to that of the pure PVA film, although the intensity of the peak at 2θ = 19.7° is lower. For films with 55% *w*/*w* glycerol (red curve), this peak does not appear in the diffractogram. The small peak at 22.5° decreases for films with glycerol compared to pure PVA. Interestingly, the peak at 2θ = 40.7° does not change substantially for the different samples studied.

It is worth noticing that for the diffraction peak at 2θ = 19.7° in the Figure 3, apparently, the ratio between the intensities corresponding to the diffraction of $\left(10\bar{1}\right)$/(101) planes changes with the addition of glycerol (see the inset in Figure 3). For the film with 22% *w*/*w* glycerol, the diffraction from $\left(10\bar{1}\right)$ planes slightly increases compared to that of the pure PVA films. A similar effect was reported in [36,39] for pure PVA films with different water content. The incorporation of water molecules in the PVA matrix induced an increase in the diffraction signal of the $\left(10\bar{1}\right)$ planes compared to that of the (101) planes. As this could be an effect of water plasticization, it is understandable that the incorporation of a small amount of glycerol also induces a similar effect. In addition, the incorporation of glycerol is expected to increase the water content in the PVA film. As more glycerol is incorporated into the film (36% *w*/*w*), the effect is reversed. The ratio of the $\left(10\bar{1}\right)$/(101) signals and the shape of the amorphous halo in the diffractogram of the PVA/glycerol 36% *w*/*w* film become similar to those of the pure PVA film.

For the quantitative description and understanding of glycerol impact on crystallinity of PVA, the diffraction scattering peaks in Figure 3 were deconvoluted into fundamental Gaussian curves with R^2^ = 0.99; 0.95; 0.98; 0.95 for 0, 22, 36, and 55% *w*/*w* glycerol, respectively [40] (Figure 3b–e), and the degree of crystallinity of the PVA films was calculated from the XRD data using the following formula [38]:

(2) $$\alpha =\frac{A_{c}}{A_{c}+A_{a}}\times 100\%$$

where *A_c_* and *A_a_* are the areas of the crystalline and amorphous phases, respectively.

The crystallinity for PVA/glycerol 55% *w*/*w* was not calculated, as the main PVA crystalline peak at 19.7° did not appear in the diffractogram, and the most prominent feature was the broad amorphous halo.

The results obtained for the different films are indicated in Table 1. As it is noticeable from Table 1, the results derived from analysis of the XRD data support those obtained from the FT-IR spectra.

Interestingly, the deconvolutions in Figure 3b–d show that the characteristic broad amorphous halo in the films of pure PVA and 22% *w*/*w* glycerol is centered at 2θ = 27° (Figure 3b,c). As discussed above, for the film with 22% *w*/*w* glycerol, the amorphous halo decreases compared to that of the pure PVA film. According to the FT-IR results, the glycerol molecules in this film are expected to be preferentially attached to the PVA chains through H bonding of their secondary alcohol. These molecules may cause the PVA chains to restructure, altering the organization of the amorphous phase and resulting in a slight increase in crystallinity. For the film with 33% *w*/*w* glycerol, the amorphous halo in the XRD is now centered at 2θ = 28°, and its intensity increases (Figure 3d). FT-IR indicates that as the amount of glycerol in the films increases, additional types of glycerol/PVA interactions occur. This may again lead to a restructuring of the amorphous phase and possibly the emergence of a new amorphous phase formed by aggregates of glycerol or PVA-glycerol complexes. Eventually, for the film with 55% *w*/*w* glycerol, the amorphous halo appears centered at 2θ = 33° and is prominent in the diffractogram (Figure 3e). The displacement of the amorphous halo to higher scattering angles, indicative of preferentially smaller interatomic distances in the amorphous phase, strongly supports the hypothesis that a different amorphous phase than this in pure PVA is responsible for the X-ray scattering in this case. The FT-IR data discussed above are consistent with the presence of a new glycerol-related phase in the films with 55% *w*/*w* glycerol.

In order to further study the relation between the XRD pattern and the crystallite size, the well-known Debye–Scherrer formula was used:

D = Kλ/βcosθ(3)

where D is the mean crystallite size in the direction perpendicular to the lattice planes of the selected diffraction peak, λ is the X-ray wavelength, β is the full width at half maximum (FWHM) of the peak, K is the Scherrer constant with a value of about 0.9, and θ is the diffraction angle corresponding to the selected peak in the XRD pattern. The values for D estimated from the peak at 2θ = 19.7° are displayed in Table 1.

From Table 1, it is also observed that both the degree of crystallinity and the crystallite size are slightly higher for the film of PVA with 22% *w*/*w* glycerol than for this of pure PVA. For 36% *w*/*w* glycerol, the degree of crystallinity is smaller, even though the size of the existing crystals does not experience significant modifications.

A decrease in crystallinity as a result of the addition of glycerol in PVA and PVA/starch films has been previously reported [17,41]. Also, an increase in crystallinity in PVA for low glycerol concentrations was observed by XRD [42].

The effect of glycerol on PVA crystallinity may be explained in terms of its influence on the interactions between the PVA chains. A small amount of plasticizer may increase the ability of the molecular chains to adopt different conformations and promote their ordering [43]. In order to prepare the PVA/glycerol films, we first prepare a 7.0 wt.% PVA stock solution by dissolving the purchased PVA granules in distilled water at 90 °C and then cooling to room temperature (see Section 2.1). Crystalline PVA nuclei are expected to form at this stage. Subsequently, distilled water and glycerol are added in different concentrations to prepare the final solutions, which are poured into Petry dishes and dried at room temperature. Increasing the amount of water may facilitate the ordering and assembly of existing PVA nuclei, and the presence of a small amount of glycerol in the solution may play a similar role. If glycerol is incorporated in higher quantities, glycerol aggregates may form; the glycerol molecules may selectively interact with the PVA molecular chains at the crystalline interface and even penetrate into the crystals, disrupting the molecular order [9,18].

TGA/DTA was performed to determine the temperature-dependent water/glycerol mass loss, the thermal stability, the glass transition temperature, and the melting point and enthalpy of fusion of the pure PVA and PVA/glycerol films.

**Figure 4:**
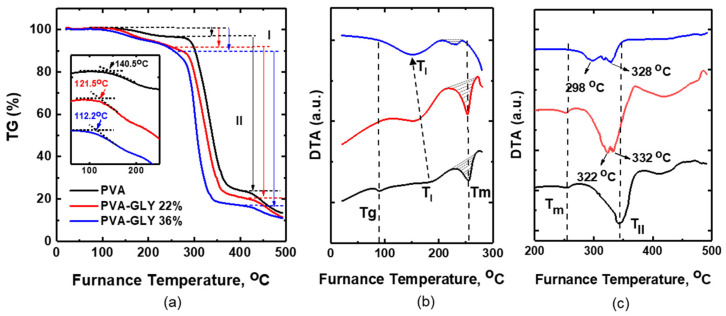
(**a**)—TG, (**b**,**c**) DTA curves of PVA (black curve) and PVA/glycerol films with 22% *w*/*w* (red curve), 36% *w*/*w* (blue curve) glycerol. The inset on (**a**) represents the onset of mass loss.

Figure 4 shows the thermogravimetric curves (mass loss versus temperature) (TG) (Figure 4a) and DTA curves (Figure 4b,c) for the PVA and PVA/glycerol films. Thermal analysis was carried out in a static air atmosphere at a 25 °C/min heating rate. The inset in Figure 4a represents the determination of the onset of mass loss for the studied samples (the curves at the inset have been arbitrarily shifted on the y-axis for ease of observation). The temperature at the onset decreases with an increase in glycerol content from 140.5 °C for PVA to 112.2 °C for 36% *w*/*w* glycerol.

The thermal transformations of pure PVA films typically occur in 4 main stages of mass loss [44,45]. The first stage corresponds to the loss of physically absorbed water, which is related to an endothermic peak in the DTA curve labeled as T_I_ in Figure 4b. The second corresponds to the partial dehydration of PVA, accompanied by polyene formation, and is related to the endothermic peak in the DTA curve labeled as T_II_ in Figure 4c. The third and fourth degradation stages are related to polyene decomposition and thermo-oxidation of carbonized residues. The degradation mechanism of PVA is explained in [46].

Table 2 lists the weight loss percentage and temperatures of the DTA peaks (T_I_ and T_II_) for the first two stages, as derived from Figure 4a–c for each sample.

The first degradation stage (Figure 4) is characterized by a weight loss of about 3.6–10.9% (Table 2), presumably related to the removal of water. According to FT-IR results, the water amount increases with an increase in glycerol content (see Figure 2b, the intensity of the peak at 1655 cm^−1^ increases). In our case, the weight loss on the first stage for PVA/glycerol films is higher than for pure PVA. However, the weight loss in this case cannot be straightforwardly attributed solely to water loss. According to [47], the thermal degradation of pure glycerol in air is characterized by a single event that takes place at 194–246 °C for a heating rate of 10 °C/min. As the first stage for PVA/glycerol 22 and 36% *w*/*w* finishes at about 270–250 °C, respectively, already above the degradation temperature of glycerol, we understand that the observed weight loss in the first stage should be due not only to water loss but also due to degradation (or initial degradation) of glycerol. As per the studies in [48,49,50], the weight loss observed in the temperature range of 125–290 °C for glycerol plasticized polymer films can be attributed to the degradation of glycerol and the loss of chemisorbed water.

The second degradation stage for the pure PVA and PVA/glycerol films is characterized by the highest weight loss. It is noticeable from Figure 4c that for the PVA/glycerol films, T_II_ shifts to lower temperatures as the glycerol content is increased. For glycerol-containing films, this peak splits into two peaks, which become more distinct for 36% *w*/*w* glycerol (298 °C and 328 °C, Figure 4c). This may be related to the degradation of a glycerol-based phase in the PVA matrix, possibly consisting of glycerol/PVA complexes, which degrade at a different temperature than pure PVA in this degradation stage.

Considering the trend for the various PVA/glycerol samples in Figure 4 and Table 2, the temperatures corresponding to the onset of mass loss and the endothermic minima of the DTA peaks T_I_ and T_II_ shift to lower values as the glycerol concentration increases. This indicates that the increase in glycerol content decreases the thermal stability of the PVA/glycerol films.

Focusing on Figure 4b, an endothermic minimum at 90 °C can be observed on the DTA curve for pure PVA. This peak, labeled Tg, is attributed to the glass transition temperature of PVA [51,52]. This peak was not observed in the DTA curves of the PVA/glycerol films.

The endothermic peak labeled T_m_ in Figure 4b can be related to the melting points of pure PVA and PVA/glycerol films [13,53,54]. Interestingly, the incorporation of 22% *w*/*w* glycerol did not lead to a significant reduction in the PVA melting point. This peak shifts to lower temperatures as the glycerol content in the film is increased to 36% *w*/*w* [13,54], indicating that the ordered association of the PVA molecules decreases with higher amounts of glycerol. The enthalpy of melting, represented by the area under the curve corresponding to the melting peak (Figure 4b), increases slightly (by ~1.6 times) for the film with 22% *w*/*w* glycerol content compared to that of the pure PVA films and decreases (by ~3.5 times) for 36% *w*/*w*. This provides further evidence of the increase and decrease in crystallinity in those samples, consistent with the FT-IR and XRD studies.

**Figure 5:**
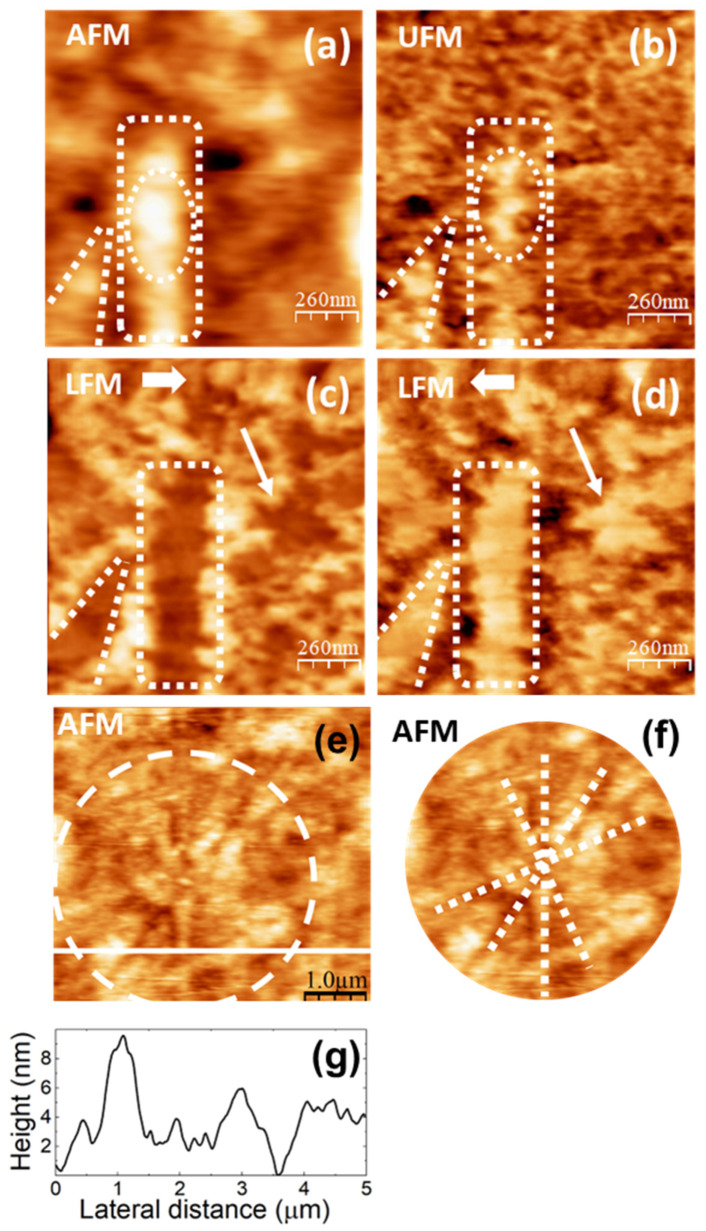
SPM images recorded on pure PVA. (**a**) Contact-mode AFM topography. Color-scale range: 8 nm. (**b**) UFM image recorded simultaneously with (**a**). (**c**,**d**) LFM images recorded over the same surface region than (**a**) scanning from left to right 🡆 (**b**) and from right to left 🡄 (**c**). (**e**) Contact-mode topographic image. Color-scale range: 16 nm. (**f**) Region enclosed by the dashed white circle in (**e**). (**g**) Height-contour profile over the continuous white line in (**e**).

**Figure 6:**
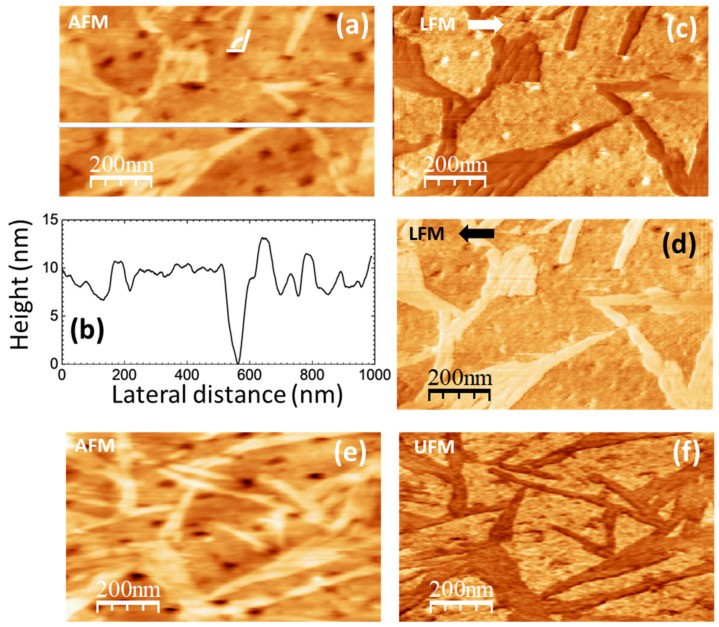
SPM images recorded on pure PVA. (**a**) Contact-mode AFM topography. Color-scale range: 17 nm. (**b**) Height-contour profile over the continuous white line in (**a**). (**c**,**d**) LFM images recorded over the same surface region than (**a**) scanning from left to right 🡆 (**c**) and from right to left 🡄 (**d**). (**e**) Contact-mode AFM topographic image. Color-scale range: 17 nm. (**f**) UFM image recorded simultaneously with (**e**) over the same surface region.

SPM images of the pure PVA films are displayed in Figure 5 and Figure 6. They were obtained from different pieces cut from the same sample cast on a 90 mm Petri dish, and, as will be explained in detail below, we understand that they illustrate different stages—possibly determined by the rate of water evaporation—of the development of crystalline domains in the semicrystalline PVA film.

In Figure 5a, the topography is characterized by clusters of aggregates, some of which appear aligned along a well-defined direction, like those enclosed within the white dashed lines. In the UFM image, recorded over the same surface area (Figure 5b), the shape of the clusters that form the topographic aggregates is much better resolved. A close look into the area outlined by the white dashed ellipse in Figure 5b reveals the presence of clusters ~45 nm in diameter, which yield a higher (stiffer) UFM contrast, forming a zig-zag chain. In the topographic image (Figure 5a), the cluster size appears to be ~80–100 nm, which may be due to UFM detecting the stiffer zones within the individual clusters. Most of the clusters within the white dashed lines in Figure 5b exhibit a stiffer contrast. In LFM (Figure 5c,d), aggregates within those dashed lines are imaged with a lower frictional contrast. Notice that in Figure 5c,d, other areas, like, for instance, that indicated by the white slanted arrows, give rise to a similar lower frictional contrast, which now does not directly correlate with any particular features of the topographic and/or UFM contrast.

We conjecture that the alignment of PVA clusters along well-defined orientations observed in Figure 5 originates from a correlated growth or assembly of PVA crystallites within the amorphous PVA matrix. Semicrystalline polymers typically crystallize into lamellar structures where molecular chains fold into layers. Spherulitic crystal growth begins with a primary nucleus at the center, from which radial fibrils consisting of individual lamellar structures separated by amorphous material radiate outwards [55]. The stiffer contrast observed in UFM for the aligned clusters is consistent with crystalline order, leading to a locally higher density in those regions. Previous studies of crystallite nucleation in PVA films with sodium montmorillonite fillers by AFM techniques support this interpretation [56]. The crystalline areas of polymer films are expected to yield a lower friction contrast, as in the case of the aligned clusters in Figure 5c,d [56]. A higher amount of water is actually expected at the PVA amorphous/crystalline interface regions [57], which may act as a lubricant, reducing friction. The areas with a similar lower frictional contrast in Figure 5c,d not correlated to specific features in the topographic or UFM image (e.g., area pointed by the white slanted arrow) may be due to an inhomogeneous distribution of water at the surface or near-surface area of the polymer films.

Figure 5e shows a larger topographic image of the PVA surface. Intersecting rows of PVA cluster aggregates are noticeable in this image. In the area within the white dashed circle in the image, some of the rows appear to stem from a primary nucleus at the center. In Figure 5f, white dotted straight lines have been drawn following the paths of some of the intersecting aligned-aggregate rows in this region to facilitate their visualization. Figure 5g corresponds to a height contour profile along the white continuous line, showing that the surface features are maintained at about 8 nm in height. The observed radial structures in the surface morphology strongly suggest incipient dendritic or spherulitic crystal growth.

Images in Figure 6 were obtained on a different piece cut from the same PVA sample cast on a 90 mm Petri dish. Here, the surface topography (Figure 6a,e) exhibits higher terraces consisting of straight ribbons ~40 nm wide, similar to the “ribbons” enclosed within the white dashed lines in Figure 5a, presumably also formed by aligned clusters as in Figure 5b, even though in Figure 6a,e the clusters cannot be distinguished. Figure 6a,e strongly resembles the images reported in [56,58,59], from which we confidently infer that the ribbon-like regions correspond to PVA crystallites. According to [58,59], the longer sides of the parallelogrammic platelets in Figure 6a should correspond to (101) planes and the shorter sides to (100) planes, being the acute angle measured from Figure 6a indeed consistent with the reported value of 55°.

The LFM images (Figure 6c,d) indicate that the ribbon-like areas yield lower frictional contrast, as expected for crystalline regions [56].

Figure 6e,f was recorded over a region similar to that of Figure 6a–c. The topography in Figure 6e shows higher ribbon-like features similar to those in Figure 6a. Nevertheless, in contrast with the results in Figure 5a,b, UFM now yields a lower (softer) contrast at the ribbon areas, and individual PVA clusters cannot be resolved.

The topographical differences between the PVA surfaces in Figure 5 and Figure 6 might be explained by a slightly different water content in the two regions. According to the XRD data, the size of the PVA crystallites is expected to be ~5 nm. Hence, several small crystalline nuclei may coexist within the clusters observed in Figure 5a,b. In Figure 5, we observed that PVA clusters align, forming rows and ribbon-like structures. In Figure 6, the ribbon-like structures appear much better defined. In view of our results, we attribute the differences between Figure 5 and Figure 6 to the fact that the sample areas on which the images were taken are at different stages of the evolution of the surface PVA crystalline structures that result from the assembly of the crystalline nuclei. The presence of water is expected to facilitate the reorganization and assembly of the PVA clusters. Hence, the formation of better-defined ribbon-like structures—presumably related to PVA crystalline domains—in the regions where Figure 6 was recorded can be explained if the local content of water in this region is slightly higher. In PVA solutions, water may be bonded to the PVA molecular chains through hydrogen bonds at hydroxyl sites (bound water) or remain as free water within the polymer matrix [60]. As PVA crystallization evolves, it is expected that bound water remains at the amorphous/crystalline interface. This may exert a plasticizing effect and lead to a softer (lower) UFM contrast at the PVA crystalline ribbons, as well as hinder the identification of individual PVA clusters at those sites.

Figure 6b depicts a height-contour profile along the line in Figure 6a, which is consistent with a multilayer structure of the surface topography with steps of ~2 nm defining the different height levels. In both Figure 5a,d and Figure 6a,e, pores are apparent. These pores, with a diameter similar to the width of the ribbons, might result from the reorganization of surface clusters during the assembly of the ribbon-like structures.

**Figure 7:**
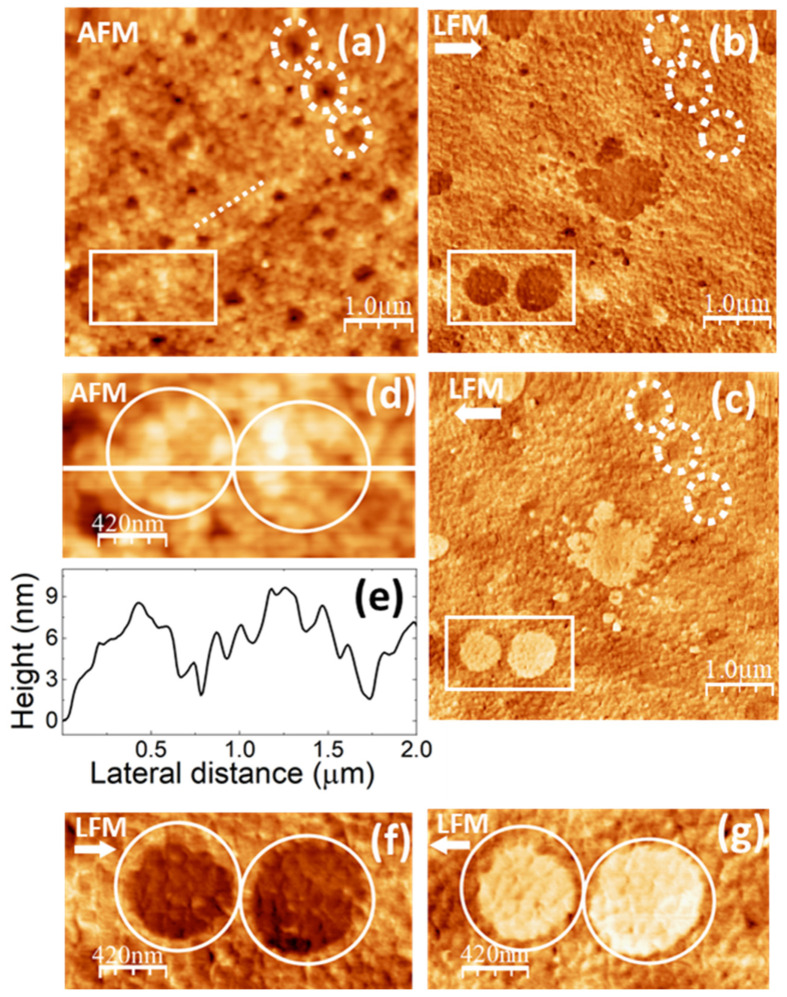
SPM images recorded on PVA/glycerol 22% *w*/*w*. (**a**) Contact-mode AFM topography. Color-scale range: 26 nm. (**b**,**c**) LFM images recorded over the same surface region than (**a**) scanning from left to right 🡆 (**b**) and from right to left 🡄 (**c**). (**d**) Enlargement of the region enclosed by the white rectangle in (**a**). (**e**) Height-contour profile over the while line in (**d**). (**f**,**g**) Enlargements of the regions enclosed by the white rectangles in (**b**,**c**).

Figure 7 shows SPM images of the PVA/glycerol 22% *w*/*w* film. The size of the AFM topographic image in Figure 7a is the same size as this in Figure 5e. In Figure 7a, the topography is also characterized by cluster aggregates with a size similar to those on the pure PVA film surface (~100 nm as measured in the contact-mode AFM topographic image). Also, in this image some of the clusters are aligned (e.g., those beside the dashed line on the image), even though the length of the clusters row is considerably shorter.

The LFM images (Figure 7b,c) indicate here the presence of areas with a lower frictional contrast, similar to those in Figure 5c,d and Figure 6c,d, although now those are not ribbon-like or parellogrammic in shape but rather rounded. Such areas do not appear straightforwardly correlated to specific topographic features.

Figure 7d corresponds to an enlargement of the topographic area enclosed by the rectangle in Figure 7a. Figure 7e displays a height-contour profile along the straight line in Figure 7d. Figure 7f,g shows enlargements of the LFM images of the same surface area, also enclosed by rectangles in Figure 7b,c, respectively. The rounded areas with a lower frictional contrast have been enclosed by circles in Figure 7f,g, and circles over the same area have also been drawn in Figure 7d to facilitate the analysis of the topography on those areas. In both the topography and the LFM images, clusters may be clearly distinguished in the regions enclosed by the circles. However, clusters that appear topographically identical do not produce the same frictional contrast. In Figure 7d, we observe clusters located at different layers. A detailed examination of Figure 7a–c allows us to infer that at the topographic regions around those that yield a lower frictional contrast, the clusters tend to be located at higher sites. It is also interesting to remark that in Figure 7f,g, the boundary regions of the lower friction domains appear defined by individual clusters, as is also the case in Figure 5c,d. This supports the interpretation that the assembly of the PVA crystalline domains proceeds through the assembly of the PVA clusters themselves.

We hypothesize that the areas with distinct frictional contrast in Figure 7b,c,f,g correspond to crystalline PVA domains, whose shape has been modified by the incorporation of 22% *w*/*w* glycerol in the PVA matrix. According to our preparation method, we first prepare a 7.0 wt.% PVA stock solution, which we then dilute to 3.5 wt.% PVA by adding a glycerol/water solution at the appropriate concentration. The addition of the glycerol/water solution is carried out at room temperature (25 °C). We presume that the initial PVA crystalline nuclei are already present in the 7.0 wt.% PVA stock solution. These nuclei would have formed as the solution was slowly cooled from 90 °C to room temperature. The growth or assembly of these nuclei is expected to take place as the resulting solution is eventually dried. Glycerol will incorporate into the PVA chains through hydrogen bonds, but if only in a small quantity, it will most likely not penetrate inside the PVA crystallites. Instead, it will preferentially accumulate at the amorphous/crystalline interface, as it happens with water. The incorporation of a small amount of glycerol into the PVA molecules may help the assembly of the crystalline domains, fostering the reorganization of the amorphous PVA regions. This would explain the fact that the amorphous halo observed in the XRD diffractogram (Figure 3) diminishes for the PVA/glycerol 22% *w*/*w* films and the fact that the crystallinity slightly increases as inferred from the XRD, FT-IR, and DTA data. In addition, the modification of the shape of the PVA crystalline domains allows us to explain the changes in the relative intensities of the XRD peaks.

The presence of numerous pores is also noticeable in Figure 7a. As in Figure 6a, many of those pores appear to be originated by a missing PVA cluster. Pores with a larger diameter, which might correspond to the displacement of various neighboring clusters, may also be seen in the topographic image. For instance, those within the white dotted circles in Figure 7a have a diameter of ~300 nm. Notice that those pores produce a higher frictional contrast in the LFM images, in which the same locations have also been marked with white dotted circles to facilitate their localization. The origin of this contrast will be discussed in detail below.

**Figure 8:**
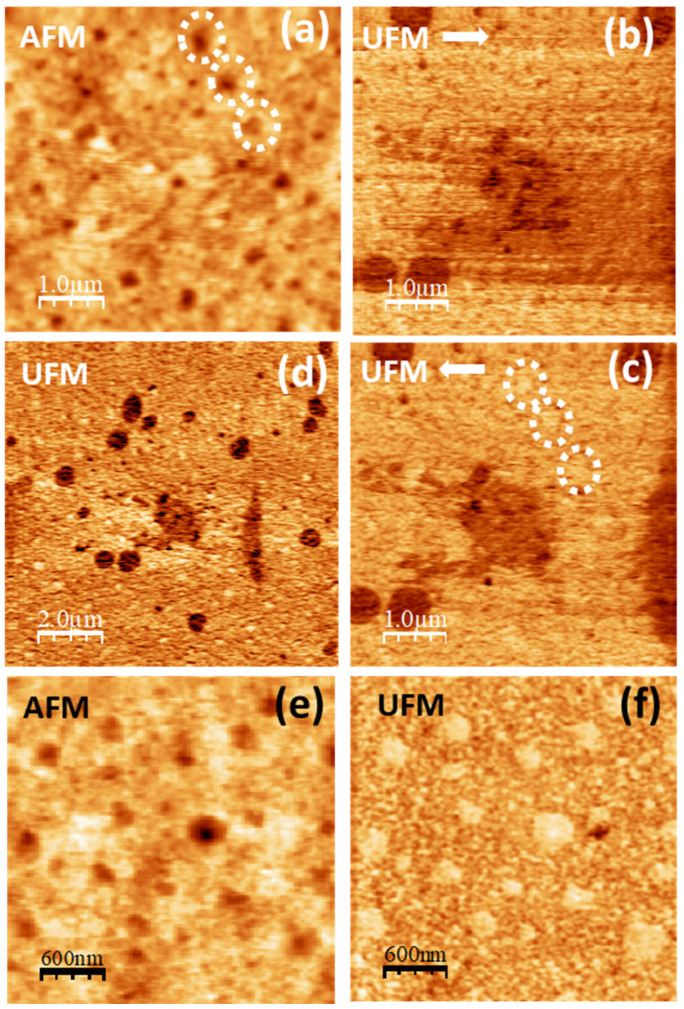
SPM images recorded on PVA/glycerol 22% *w*/*w*. (**a**) Contact-mode AFM topography over the same surface region as Figure 7a. (**b**) UFM image recorded simultaneously with (**a**), scanning from left to right 🡆. (**c**) UFM image recorded together with (**a**) but scanning from right to left 🡄. (**d**) UFM image over a larger area than (**c**), centered at the same position. (**e**) Contact-mode AFM topography at nearby region. Color-scale range: 24 nm. (**f**) UFM image recorded simultaneously with (**e**).

In Figure 8, also referred to as the PVA/glycerol 22% *w*/*w* film, the topography (Figure 8a) and the simultaneous UFM image (Figure 8b) were recorded over the same surface region as Figure 7a–c. The three pores are enclosed by the white dotted circles in the upper right corner in both Figure 7a and Figure 8a and allow the identification of the regions slightly displaced from each other due to drift. Figure 8a,b was recorded with the tip scanning forwards (from the left to right), and Figure 8c was recorded together with Figure 8b with the tip scanning backwards (from right to left). As it is apparent from Figure 8b,c, while scanning from left to right in the presence of ultrasonic vibration, some of the PVA surface clusters from the right-hand side were dragged to the left by the tip and remained there as the tip scanned back to produce Figure 8c. A close look at Figure 8a shows straight features resulting from the dragging of the clusters, even though the topography does not seem to be severely affected by the tip-induced modifications. In Figure 8c, we find areas with a lower (softer) UFM contrast in similar locations as those that in Figure 7b,c yielded lower frictional contrast. As it is apparent, the shape of domain located at the center in Figure 8c is markedly different from this in Figure 7b,c, presumably as a result of the tip action. At the lower right-hand border of Figure 8c, the image shows a heap with darker (softer) contrast. Figure 8d corresponds to a larger UFM image recorded over a region that included the area of Figure 8a–c in its central part, recorded following the latter. The heap with lower UFM contrast induced by the tip while producing Figure 8b can be clearly distinguished here. Such heap presumably consists of clusters drugged by the tip while scanning forwards and piled up at the end of the scan. Interestingly, some of the other domains in Figure 8a–c, like those with a rounded shape at the lower right-hand corner of the image, remained unaffected.

When discussing Figure 7a–c we identified the lower friction domains as PVA crystalline regions. The fact that those domains yield a lower (softer) UFM contrast may be explained because of the presence of glycerol (and possibly also water) at the amorphous/crystalline interface areas. UFM images in Figure 6f also yielded a lower UFM contrast in the crystalline domains of the pure PVA films. However, we cannot rule out that in PVA/glycerol films, clusters incorporating glycerol molecules produce a softer and lower frictional contrast even if they do not contain crystalline nuclei.

Glycerol molecules located at PVA cluster surfaces may facilitate the displacement of those clusters relative to each other, and this effect may certainly be enhanced by the presence of surface ultrasonic vibration, promoting tip-induced modifications while scanning.

In addition, the glycerol molecules might favor the detachment of surface clusters and induce surface pores. The simultaneous topographic and UFM images in Figure 8e,f show an area in which various surface pores of different sizes up to ~300 nm are apparent. As can be seen in the images, the UFM contrast is stiffer (brighter) within the pores. Moreover, as it was mentioned above, a higher frictional contrast is also found within pores (see Figure 8a–c, pores located within the white dashed circles). The size of the pores rules out that this contrast is an artifact. In fact, such contrast is not observed in the pores found in the pure PVA films (see Figure 5). The presence of pores on the PVA/glycerol 22% *w*/*w* films and the UFM and LFM contrast at the pores’ location may indicate that PVA clusters located at the bottom of the pores have incorporated glycerol molecules on their surfaces. On the one hand, this may facilitate the detachment of the clusters at the top, explaining the formation of the pore itself. On the other hand, glycerol molecules in a cluster bonded with their primary alcohol groups to a PVA molecular chain and with their secondary alcohol groups to a neighboring PVA molecular chain could exert a hydrogen bond-mediated crosslinking effect on PVA, increasing the stiffness of the confined cluster [11].

**Figure 9:**
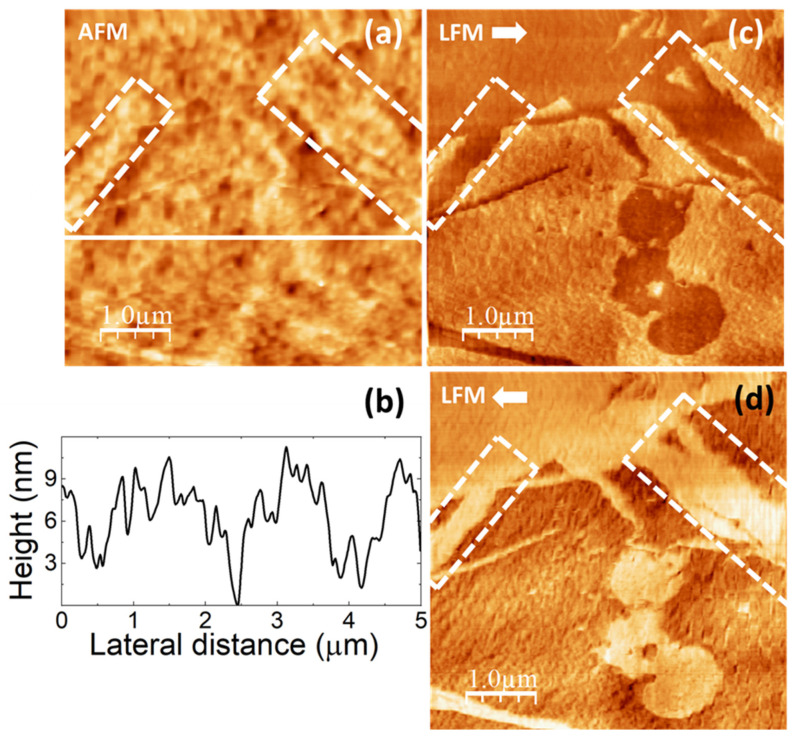
SPM images recorded on PVA/glycerol 36% *w*/*w*. (**a**) Contact-mode topography. Color-scale range: 28 nm. (**b**) Height-contour profile recorded along the continuous white line in (**a**). (**c**,**d**) LFM images recorded over the same surface area as (**a**), scanning from left to right 🡆 (**c**) and from right to left 🡄 (**d**).

Figure 9 shows SPM images of the PVA/glycerol 36% *w*/*w* film. Figure 9a corresponds to a contact-mode AFM topographic image, recorded over an area of the same size as Figure 5e and Figure 7a. As in the case of the pure PVA and the PVA/glycerol 22% *w*/*w* film, the topography is characterized by cluster aggregates, some of which are aligned into rows. Figure 9b displays a contour-line profile along the straight line in Figure 9a.

Figure 9c,d corresponds to lateral force microscopy images scanning from left to right (Figure 9c) and from right to left (Figure 9d) recorded over the same surface region as (Figure 9a). As it is clearly seen in Figure 9c,d, we find areas with a lower frictional contrast, similar to those in Figure 5c,d, Figure 6c,d, and Figure 7b,c. While some of those areas exhibit rounded interfaces similar to those in Figure 6c,d, others rather resemble the ribbon-like shape domains found in the pure PVA film surface (Figure 5c,d and Figure 6c,d). Nevertheless, here, the extension of the lower contrast domains is apparently larger, as if some of the areas linking the domains in Figure 6 that for the pure PVA produced a higher friction contrast, were now also yielding a low frictional response. Interestingly, some of the ribbon-like low friction domains in Figure 9c,d (like, for instance, those within the white dashed rectangles) seem to be correlated to higher topographic regions, but at other areas of the low friction domains, no correlation with the topographic image could be appreciated.

According to the FT-IR and XRD data, the crystallinity of the PVA/glycerol 36% *w*/*w* films is reduced compared to this for the pure PVA and 22% *w*/*w* glycerol films. This may be explained by the formation of glycerol aggregates for the higher glycerol concentrations, which may penetrate within the crystalline PVA domains and partially destroy them. For these films, in the DTA curves, the endothermic peak corresponding to the maximum rate of degradation for PVA appears split into two well-resolved peaks, which might be indicative of the formation of a new phase formed by glycerol aggregates or glycerol/PVA complexes. The lower friction contrast in Figure 9b,c might originate either because of the presence of crystalline PVA domains, or of the presence of a distinct phase formed by glycerol/PVA complexes, even though the LFM image provides no clue to justify the presence of lower frictional domains of a different nature.

It should be pointed out that on the PVA/glycerol 36% *w*/*w* films, we did not succeed in recording good quality UFM images, as the film was severely affected by tip actuation in the presence of ultrasound. And for the case of PVA/glycerol (55% *w*/*w*), nor even contact AFM and LFM images could be recorded without surface modification.

According to FT-IR, XRD, and DTA data, the incorporation of 55% *w*/*w* glycerol leads to a significant reduction in the PVA crystallinity. The effect may be similar to this observed with water, which in a small amount enhances the PVA crystallinity, but excess water leads to destruction of the PVA crystallites.

## 4. Conclusions

The study presents a thorough investigation of the structural properties, crystallinity, and thermal response of pure PVA and PVA/glycerol films using FT-IR, XRD, TG/DTA, and advanced SPM. The combination of different types of SPM techniques (contact mode AFM, LFM, and UFM) allowed us to obtain information on the nanoscale topography and frictional and mechanical response of the films’ surface.

In the pure PVA films, SPM allowed us to observe ribbon-shaped domains and clusters aligning along a well-defined direction and forming intersecting rows with a different frictional and elastic contrast, which apparently originated from a correlated growth or assembly of PVA crystalline nuclei (~5 nm in diameter according to XRD) located within individual PVA clusters (~80–100 nm in diameter). The incorporation of 22% *w*/*w* glycerol leads to a modification in shape of those domains from ribbon-like in pure PVA to rounded in the 22% *w*/*w* PVA/glycerol films. In correlation with this, the XRD patterns show changes in the relative intensities of the XRD crystalline peaks and a decrease in the amorphous halo, while the melting point remained almost the same. In addition, pores of various sizes up to 300 nm in diameter were observed, which produced a characteristically higher frictional and higher (stiffer) UFM contrast. At the film with 36% *w*/*w* glycerol content, domains with lower frictional contrast were also observed, some with rounded interfaces like those seen in PVA/glycerol 22% *w*/*w* films, and others that rather resembled the ribbon-like domains found in the pure PVA film surface but were wider and joined together. For these films, the amorphous halo in the XRD diffractogram increases and slightly displaces to higher 2θ angles. The observation of larger lower friction domains in this case may also originate from a new phase formed by glycerol aggregates or glycerol/PVA complexes, even though LFM does not provide any clue to justify a different nature of coexisting lower friction domains. The aforementioned changes are accompanied by a decrease in crystallinity to values of pure PVA according to FT-IR and XRD and a decrease in melting point. For glycerol contents higher than 36% *w*/*w*, tip-induced modifications severely complicated the acquisition and analysis of SPM images. FT-IR revealed additional glycerol-characteristic peaks presumably related to the formation of glycerol aggregates; the amorphous halo was prominent in the XRD diffraction pattern and was clearly displaced to a higher 2θ, and both XRD and FT-IR confirmed a drastic reduction in crystallinity.

According to the obtained results, glycerol molecules in a small quantity do not penetrate inside the PVA crystallites but foster the reorganization of the amorphous PVA regions, alter the shape of the PVA crystalline domains, and preferentially accumulate at the amorphous/crystalline interface. In the PVA/glycerol films, the domains could only be clearly identified from LFM and UFM; no topographical correlation was apparent. As the glycerol content increases, glycerol begins to interact with PVA within the crystalline structures, being those nearly completely disrupted for high glycerol contents. The mobility of PVA chains increases substantially, and tip-induced surface modification effects were observed, which were more significant in the presence of ultrasonic vibration.

The presented research contributes to the understanding of PVA crystallinity and its modification by plasticizers such as glycerol. Our findings deliver crucial insights into how glycerol impacts that nanostructure of PVA, thereby paving the way for tailoring PVA properties to achieve optimal performance.

## Figures and Tables

**Table 1:** Crystallinity percentage (a) of PVA films with different glycerol content calculated by FT-IR (Figure 2) and XRD (Figure 3, Debye–Scherrer formula applied to peak at 19.7°); melting points (T_M_) determined from DTA (Figure 4c).

Glycerol (% *w*/*w*)	α(FT-IR)	α(XRD)	Crystal Size (nm)	T_M_ (°C)
0	27.1	27	4.25	255
22	30.4	30	5.08	253
36	27.3	25	4.90	231
55	19.5		-	-

**Table 2:** Weight loss and temperatures corresponding to maximum rate of weight loss at first two stages (determined in Figure 4a–c).

Sample	Degradation Step	Weight Loss, %	T_DTA peak_, °C
PVA	I	3.6	186
	II	72.9	342
PVA-GLY 22%	I	8.6	161
	II	71.4	332/322
PVA-GLY 36%	I	10.9	150
	II	72.4	328/298

## Data Availability

The original contributions presented in the study are included in the article, further inquiries can be directed to the corresponding author.
